# Case report: Primary mediastinal Ewing’s sarcoma presenting with chest tightness

**DOI:** 10.3389/fonc.2022.1020339

**Published:** 2023-02-06

**Authors:** Manman Cui, Duchang Zhai, Yan Liu, Xiuzhi Zhou, Tingting Wang, Lihuan Wang, Wu Cai, Guohua Fan, Shenghong Ju

**Affiliations:** ^1^ Department of Radiology, The Second Affiliated Hospital of Soochow University, Suzhou, China; ^2^ Department of Radiology, The First People`s Hospital of Taicang, Taicang, China; ^3^ Department of Radiology, Zhongda Hospital, Medical School of Southeast University, Nanjing, China

**Keywords:** Ewing’s sarcoma, extraskeletal, oncology, mediastinum, primary, chest

## Abstract

Ewing’s sarcoma is a part of a rare group of malignant neoplasms, whose pathological morphological features are small round cells. Extraskeletal Ewing’s sarcoma is a more uncommon primary tumor. Herein, we report the case of a 66-year-old man who complained of chest tightness. Subsequent chest CT scans revealed an irregular and uneven density mass on the right side of the anterior mediastinum with invasion of the superior vena cava, pericardium and right lung. The patient’s clinical symptoms were improved after performing excision of the mediastinal lesions under cardiopulmonary bypass. Based on histological and immunohistochemical findings, the tumor was diagnosed as extraskeletal Ewing’s sarcoma.

## Introduction

Extraskeletal Ewing’s sarcoma (EES) is a small round cell malignant tumor that grows outside of bone tissue, which arises from the mediastinum is extremely rare. Compared with patients with bone tumors, patients with EES have an older average age of onset. The clinical features and imaging appearances are non-specific, and patients often present with a rapidly growing mass that causes localized pain (1). The diagnosis of this tumor mainly relies on pathological, cytogenetic, immunohistochemical and molecular genetic analysis. We report a patient who presented with chest tightness and had a mediastinal ES, which was not confirmed until postoperative pathological examination was performed.

## Case report

**Figure 1:**
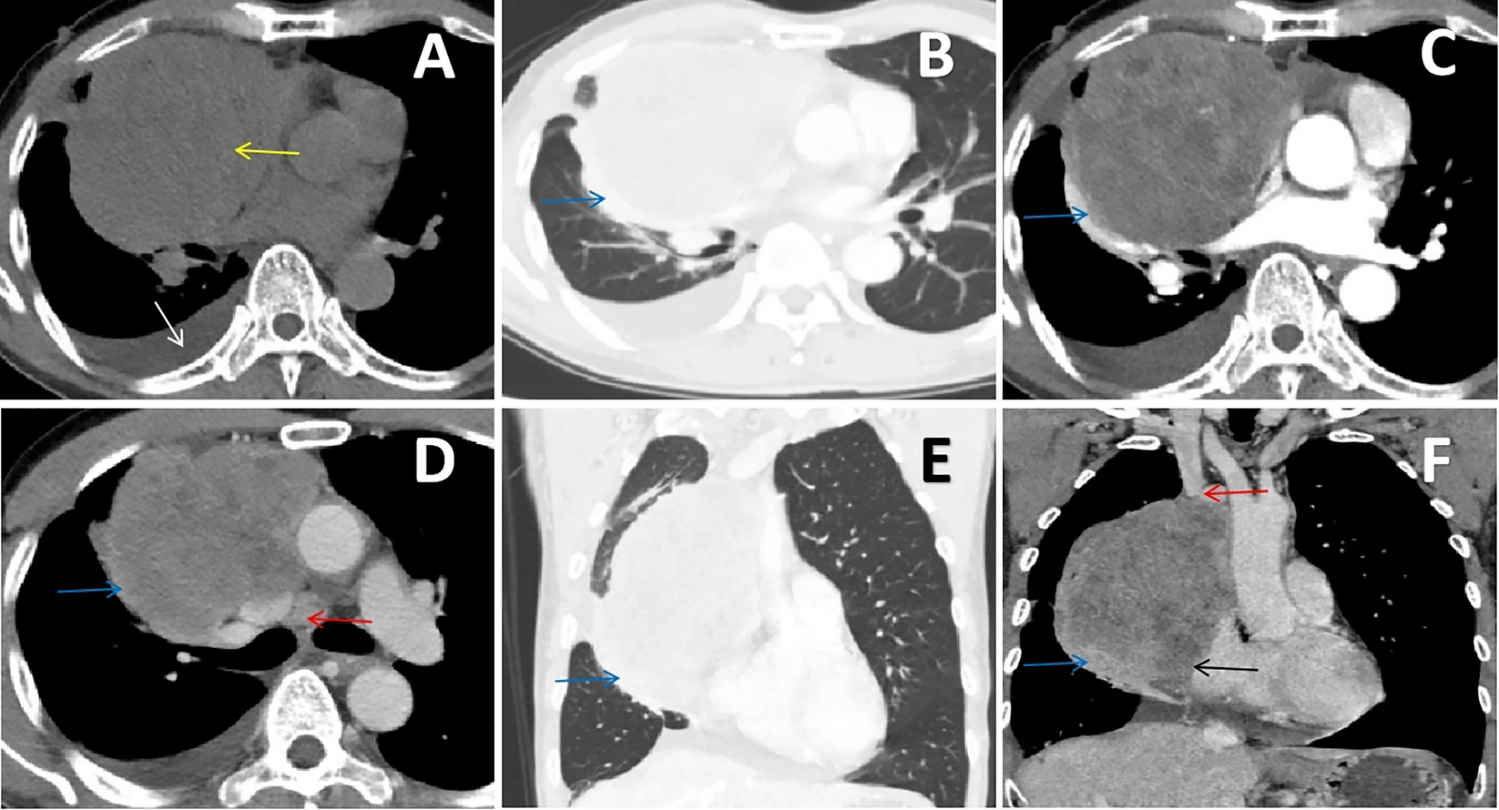
**(A, B)** Chest CT scan showing an irregular soft tissue density mass (yellow arrow) on the right side of the anterior mediastinum that compressed the adjacent right lung (blue arrow), accompanied by pleural effusion (white arrow). **(C, D)** Contrast-enhanced chest CT scan showing that the mass had moderately uneven enhancement, which invaded the right lung (blue arrow), and the mediastinal lymph nodes were enlarged (red arrow). **(E, F)** The coronal multiplanar reconstruction images clearly delineated the irregular and uneven density mass on the right side of the anterior mediastinum with invasion of the superior vena cava (red arrow), pericardium (black arrow) and right lung (blue arrow).

A 66-year-old man with an anterior mediastinal tumor was admitted to the hospital for surgical treatment. He went to the emergency department of the hospital 1 day prior because of chest tightness, and plain chest CT showed a mass on the right side of the anterior mediastinum that compressed the adjacent right lung, accompanied by pleural effusion (**Figures 1A, B**). Laboratory examinations revealed elevated levels of troponin, cardiac enzymes, CA125, NSE and cytokeratin, which indicated myocardial injury and abnormal cancer markers. The preoperative contrast-enhanced CT scan showed a giant cystic and solid mass on the right side of the anterior mediastinum, which had moderately uneven enhancement, and the mediastinal lymph nodes were enlarged (**Figures 1C, D**). The mass on the right side of the anterior mediastinum with invasion of the superior vena cava, pericardium and right lung could be observed from the coronal multiplanar reconstruction images (**Figures 1E, F**). In summary, we initially considered that the mass was a malignant tumor arising from the thymus.

**Figure 2:**
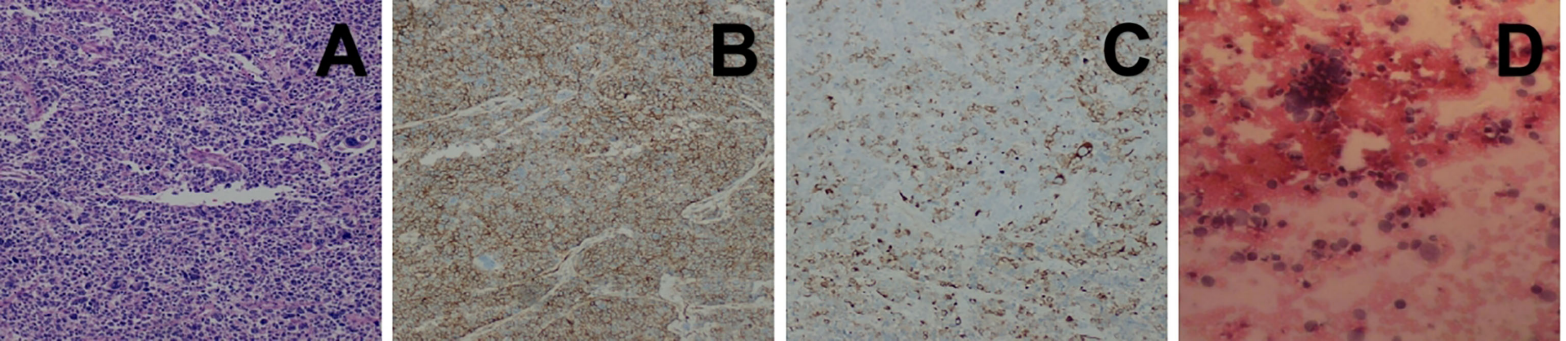
**(A)** Histopathological image showing tumor cells composed of primitive small round cells (H&E staining). **(B, C)** Immunohistochemical images showing positivity for CD99 and desmin. **(D)** Histopathological image showing pericardial effusion, which consisted of poor differentiation of malignant tumors.

Intraoperative exploration revealed that the tumor had invaded the superior vena cava, pericardium and right middle lung, so the surgeon adopted resection of the mediastinal lesions under cardiopulmonary bypass, partial resection of the superior vena cava with artificial blood vessel replacement and lobectomy of the right middle lung with lymph node dissection. The pathological findings confirmed that it was a small round cell malignant tumor (**Figure 2A**). The mediastinal lymph nodes submitted for examination showed that metastasis of the tumor had already occurred. The immunohistochemical results demonstrated a Ki‐67 of 90%, positivity for AE1/AE3, P16, CD 99 (**Figure 2B**), CD56 and synaptophysin, partial positivity for EMA, MDM2 and desmin (**Figure 2C**), and negativity for vimentin, CK7, CAM5.2, SMA, NSE and S-100. Based on these findings, primary mediastinal ES was diagnosed. The patient’s chest tightness and overall condition improved after the operation, and he was discharged after 1 month of symptomatic and supportive treatments. One month later, the patient was admitted to the hospital again. His blood test showed a significant increase in the heart failure index score. After symptomatic treatments, the symptoms improved. Subsequently, the patient’s chest tightness was not relieved, and he had orthopnea. After consultation with experts, he was considered to have postoperative cachexia of a malignant tumor. There was a risk of respiratory cardiac arrest at any time, and the prognosis was very poor. Four days later, he was transferred to the ICU for treatment. Echocardiography showed that there was a large amount of pericardial effusion, and combined with the history of the tumor invading the pericardium, the effusion was considered to be tumor tissue. Finally, pathological examination of the pericardial effusion confirmed that it had malignant tumor cells (**Figure 2D**).

Because the circulatory failure caused by pericardial tamponade was difficult to cure, the family members of the patient finally refused further invasive surgery for the patient; only noninvasive ventilators were used to maintain the patient’s vital signs, and he had entered the end stage of clinical life.

## Discussion

EES is a rare entity with high-grade malignancy that commonly involves the soft tissues of the trunk and extremities. It is more common in the lower limbs, paraspinal, retroperitoneum and chest wall and rarely occurs in solid organs. Primary mediastinal EES is extremely rare (2, 3). Therefore, the case we present can increase our understanding of this malignant tumor. Its early clinical symptoms are mostly uncharacteristic; it usually presents as a painless mass that rarely attracts attention, and the late stage manifests as a progressive enlargement of the mass accompanied by pain and the symptoms of invaded adjacent tissues. The histological pattern of EES consists of a large number of small round cells with a uniform shape, which have a high nuclear to cytoplasmic ratio (4). In immunohistochemistry, CD99, FLI-1 and vimentin are almost expressed, and NSE and synaptophysin are expressed to varying degrees. CD99, the product of the *MIC* gene, is a relatively specific antibody of the ES family. However, CD99 expression is not specific to ES, as it can be expressed by other tumors, such as ependymoma (5). To the best of our knowledge, the translocation t(11;22)(q24;q12) is an important factor for EES diagnosis as well as differential diagnosis. Delattre et al. reported that 85% of cases were associated with the translocation t(11;22)(q24;q12), which leads to the formation of the *EWS-FLI-1* fusion gene (6). The imaging findings of EES are often nonspecific, and most CT scans show that it is a soft tissue density mass in different parts. In magnetic resonance imaging (MRI), the mass shows a moderate signal on T1WI and a moderately high signal on T2WI. Sometimes the density or signal is uneven because of haemorrhage and necrosis. It is more common to see cystic degeneration and necrosis, but calcification is rarely observed, and capsules may appear. Enhanced scans can show that a mass is uneven and that the degree of enhancement is different. Since the tumor is invasive, it tends to invade surrounding tissue structures. The CT of the patient we report clearly revealed that the tumor had invaded the superior vena cava, pericardium and lung tissue. Furthermore, enhanced scanning can show the blood supplying arteries of the mass and can also be used to evaluate the feasibility of tumor surgery and determine clinical treatment plans. Since mediastinal ES is relatively rare, it needs to be differentiated from malignant thymoma, mediastinal lymphoma, teratoma, seminoma, endodermal sinus tumor and solitary fibroma. Malignant thymoma should be considered first when a large, irregular and uneven mass appears in the anterior mediastinum, which was the reason for misdiagnosis in the study. Malignant thymomas show similar radiologic findings as EWS (7). However, malignant thymomas are much less malignant than mediastinal ES, such as they usually have no lymph node metastasis and no pleural effusion. In addition, malignant thymomas are usually accompanied by myasthenia gravis. Teratomas are often accompanied by fat, calcification, and bone and soft tissue density masses. The majority of lymphomas in the mediastinum have lymphadenopathy in the neck and other areas of the mediastinum, and the clinical symptoms are more typical. If there is a homogeneous and soft tissue density solid mass with no calcification and fat accompanied by elevated β-HCG levels, then seminoma should be considered. Enhanced scans of mediastinal endodermal sinus tumors show centripetal irregular striped and reticular blood vessels, and the surrounding irregular thick-walled ring is obviously enhanced. In addition, AFP and β-HCG levels are significantly increased in most patients. Large solitary fibromas are heterogeneous, and map-like uneven enhancement and creeping blood vessels can be observed by enhanced scanning. However, the final diagnosis depends on pathology and immunohistochemistry. Adverse prognostic factors consist of large tumors, nonsurgical treatment and regional lymph node metastasis (8). Treatment of EES includes combination therapy with chemotherapy and topical therapy delivered by surgical resection, radiation, or both (9). Covelli et al. confirmed that surgical resection alone can improve the survival rate of EES patients but also proposed that surgery combined with radiotherapy and chemotherapy is the best treatment for EES (10). The patient we report underwent surgery, but he did not receive chemotherapy or radiotherapy for economic reasons. Finally, he had a poor prognosis and entered the end stage of clinical life. Liu et al. reported a case of mediastinal ES that invaded surrounding tissues and was unresectable, but after chemotherapy, the patient’s disease was almost completely in remission with no serious side effects (11).

## Conclusion

Most cases of primary EES in the mediastinum appear as masses upon diagnosis with no specific clinical symptoms. On imaging, these tumors are usually large, enveloped, soft tissue density masses that can compress or invade adjacent tissues and easily metastasize, and an enhanced scans can show inhomogeneous moderate or significant enhancement. Imaging can be performed as a diagnostic tool, but the final diagnosis is mainly based on pathology and immunohistochemistry. In terms of treatment, surgical resection combined with chemotherapy, radiotherapy or both is the best solution.

## Data availability statement

The original contributions presented in the study are included in the article/Supplementary Material. Further inquiries can be directed to the corresponding author.

## Ethics statement

Written informed consent was obtained from the participant for the publication of any potentially identifiable images or data included in this article.

## Author contributions

MC, DZ, XZ, YL, TW, LW, GF, SJ, and WC conceived the idea for the article. MC drafted the manuscript. WC approved the final version of the manuscript. All authors contributed to the article and approved the submitted version.
